# Supplementary material for: Structural evolution of a fungal cell wall protein family for β-glucan-binding and cell separation
Source: mBio. 2026 May 6;17(6):e03535-25. doi: 10.1128/mbio.03535-25 (PMC13251364; doi:10.1128/mbio.03535-25)
Supplement: Supplemental figures and tables — Fig. S1 to S9; Tables S2 to S8. [file mbio.03535-25-s0001.docx]

**Supplemental Figure S10**

**
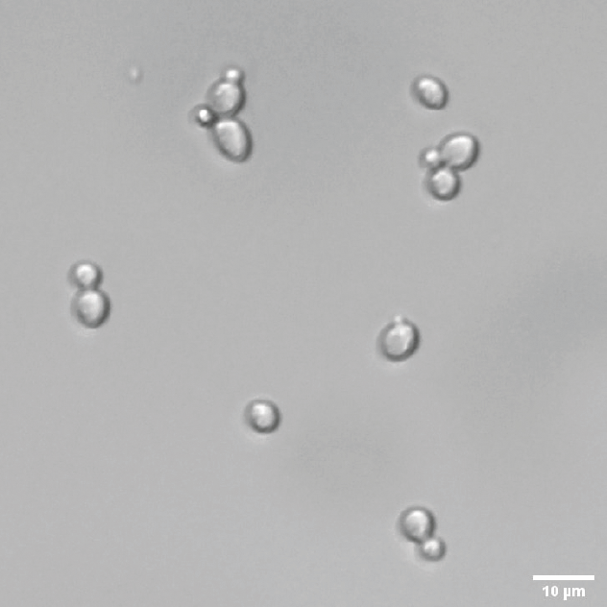

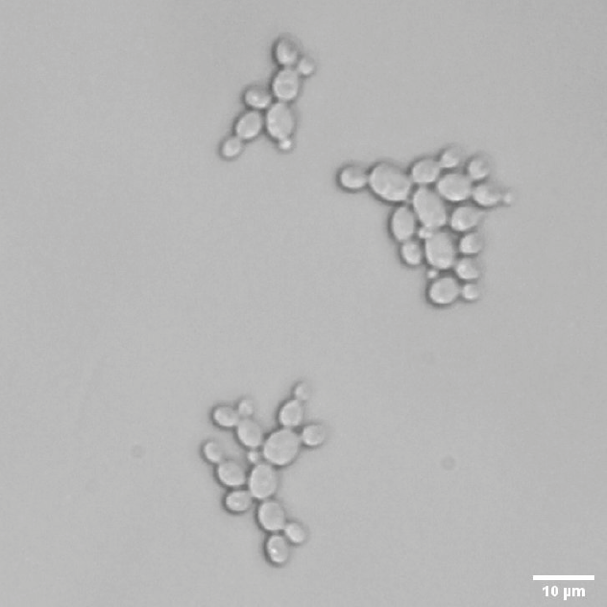
**

**
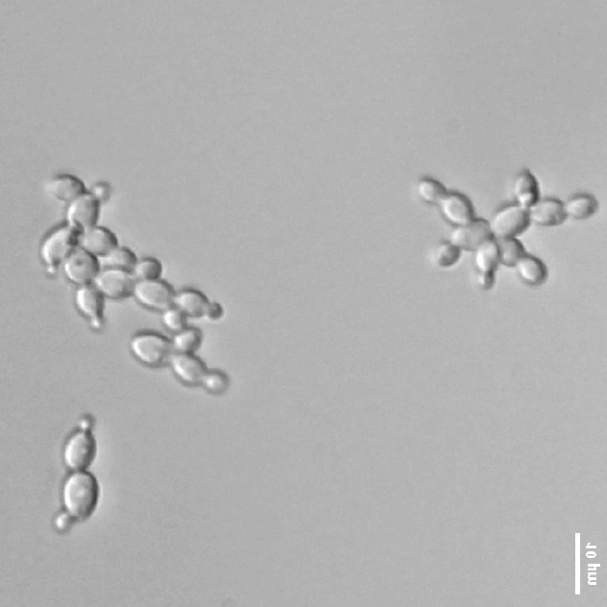

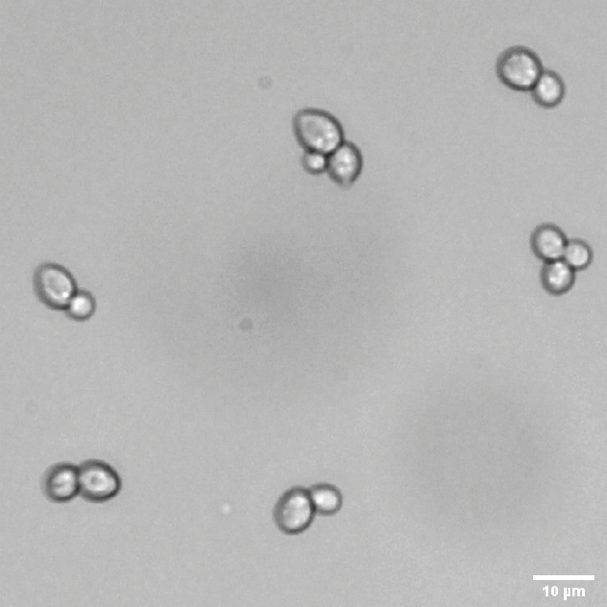
**

**Figure S10. Effect of a ScSun4 deletion mutation on cell clustering.** Microscopic images show cell clustering as analyzed by bright-field microscopy of a yeast strain (YHUM3154) carrying the chromosomal *ScSUN4* gene (top left) and a yeast strain with a chromosomal *sun4∆* mutation (YHUM3160), that was carrying either no plasmid (top right), or a control plasmid (vector pRS316) without *ScSUN4* (bottom left), or a plasmid (BHUM3438) with *ScSUN4* (bottom right), respectively. White bars corresponds to 10 µm. Supplemental Figure S10 is identical to Figure 3A, with the exception that scale bars are shown in each image.
