# Supplementary material for: Structural evolution of a fungal cell wall protein family for β-glucan-binding and cell separation
Source: mBio. 2026 May 6;17(6):e03535-25. doi: 10.1128/mbio.03535-25 (PMC13251364; doi:10.1128/mbio.03535-25)
Supplement: Figure S10 — Effect of a ScSun4 deletion mutation on cell clustering. [file mbio.03535-25-s0002.pdf]

Supplemental Figures S1 to S9

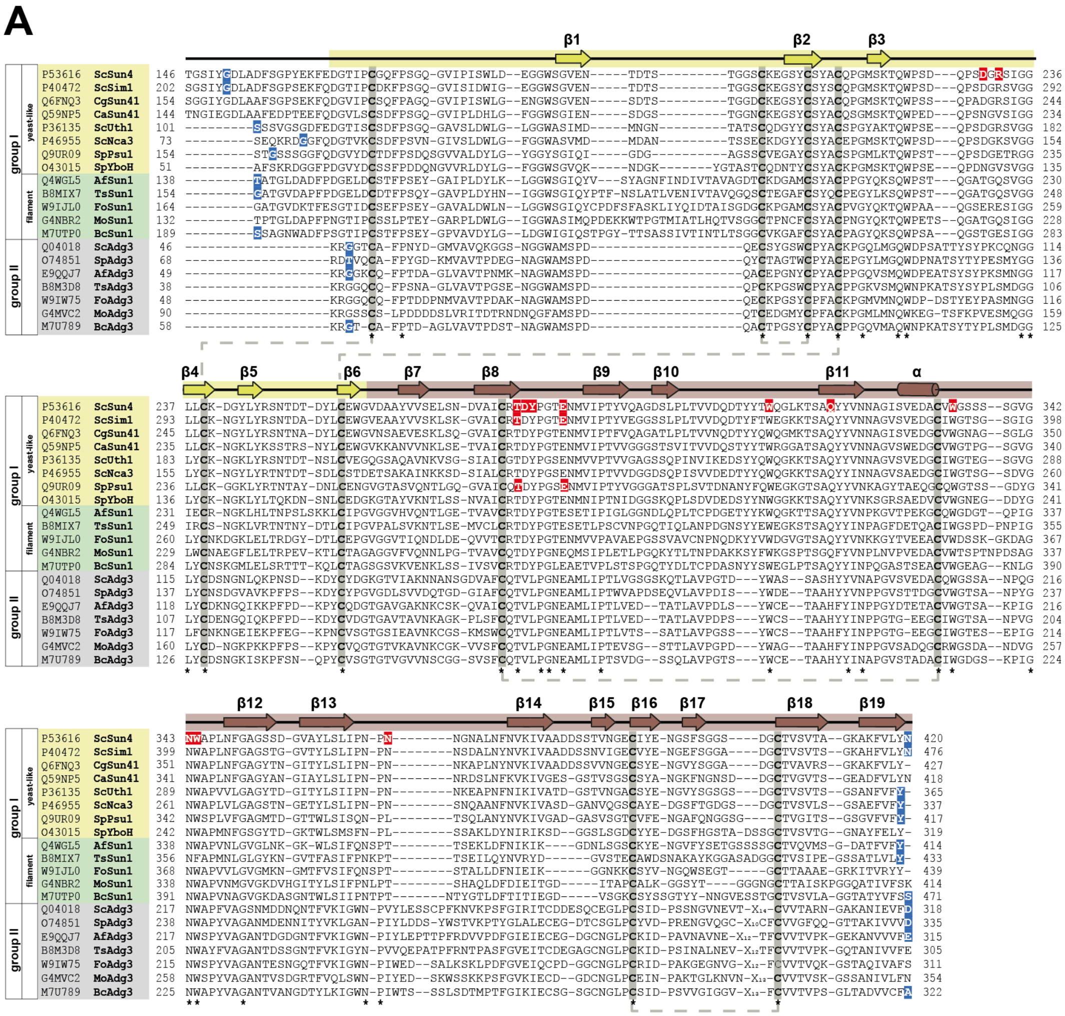

B

group	subgroup		ScSun4	ScSim1	CgSun41	CaSun41	ScUth1	ScNca3	SpPsu1	SpYboH	AfSun1	TsSun1	FoSun1	MoSun1	BcSun1	ScAdg3	SpAdg3	AfAdg3	TsAdg3	FoAdg3	MoAdg3	BcAdg3
group I	yeast-like	ScSun4	100																			
		ScSim1	85	100																		
		CgSun41	81	81	100																	
		CaSun41	70	70	70	100																
		ScUth1	62	63	61	60	100															
		ScNca3	61	64	58	58	71	100														
		SpPsu1	53	58	54	54	55	56	100													
		SpYboH	51	53	52	53	53	57	55	100												
	filament	AfSun1	43	44	43	46	48	45	48	48	100											
		TsSun1	40	44	42	42	40	38	43	40	54	100										
		FoSun1	42	41	42	40	46	41	44	41	52	46	100									
		MoSun1	36	34	36	36	39	36	33	39	48	42	47	100								
		BcSun1	39	40	40	41	45	40	40	39	50	46	49	51	100							
group II		ScAdg3	32	31	30	31	36	33	32	34	30	29	29	30	29	100						
		SpAdg3	31	31	31	32	31	33	31	30	29	30	30	31	29	54	100					
		AfAdg3	32	31	31	29	34	33	29	31	31	29	32	32	33	58	53	100				
		TsAdg3	31	31	32	31	34	33	32	26	29	30	31	30	32	60	52	72	100			
		FoAdg3	27	27	31	31	32	32	33	29	29	27	29	29	27	51	51	63	64	100		
		MoAdg3	31	29	29	32	31	31	32	31	30	27	27	27	28	50	48	56	55	60	100	
		BcAdg3	31	30	30	33	36	33	30	32	31	32	30	30	35	56	55	63	60	61	57	100

C

		group I yeast-like	group I filament	group II
group I	yeast-like	61.4		
	filament	41.1	48.5	
group II		31.3	29.8	57.1

Figure S1. Comparison of selected SUN domain sequences. A) Multiple sequence alignment of 20 fungal SUN domains covering group I and group II (according to Figure 1) as calculated by the Clustal Omega web service of the EMBL-EBI (1). For each SUN domain, the UniProt Knowledgebase ID and name of the originating protein are shown on the left side. Group I members from yeast-like fungi are highlighted in yellow and from filamentous fungi in light green, respectively. Group II members are marked in grey. Numbering of the sequences is shown according to the UniProt Knowledgebase. The regions covering the N-terminal sushi-like domain (yellow) and the C-terminal thaumatin-like domain (brown) are indicated above the sequences along with the respective secondary structure elements. The ten conserved cysteine residues are highlighted in grey and the corresponding cysteine connection pattern is shown with dotted lines. Residues that were functionally investigated by mutational analysis are highlighted in red, and residues used for construction of diverse ScSun4-chimeras (see Figure 5A) are marked in blue. B) Protein sequence identity matrix of SUN domains shown in *A)*. Numbers correspond to percentage identity values. C) Average proteins sequence identities of SUN domains of the three different groups shown in *B)*.

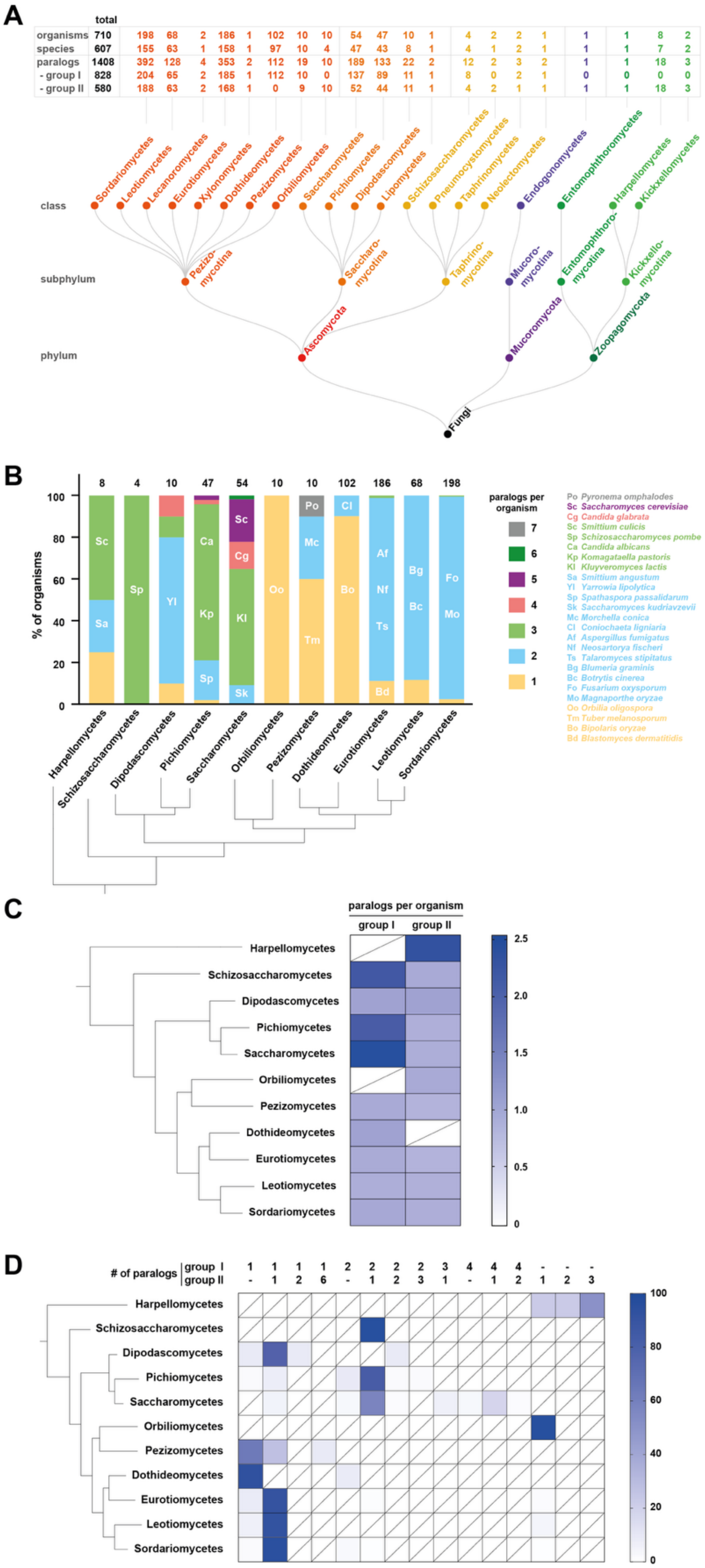
A
total
organisms 710 198 68 2 186 1 102 10 10 54 47 10 1 4 2 2 1 1 1 8 2
species 607 155 63 1 158 1 97 10 4 47 43 8 1 4 1 2 1 1 1 7 2
paralogs 1408 392 128 4 353 2 112 19 10 189 133 22 2 12 2 3 2 1 1 18 3
- group I 828 204 65 2 185 1 112 10 0 137 89 11 1 8 0 2 1 0 0 0 0
- group II 580 188 63 2 168 1 0 9 10 52 44 11 1 4 2 1 1 1 1 18 3
class
Sordariomycetes
Leotiomycetes
Lecanoromycetes
Eurotiomycetes
Xylonomycetes
Dothideomycetes
Pezizomycetes
Orbiliomycetes
Saccharomycetes
Pichiomycetes
Dipodascomycetes
Lipomycetes
Schizosaccharomycetes
Pneumocystomycetes
Taphrinomycetes
Neolectomycetes
Endogonomycetes
Entomophthoromycetes
Harpellomycetes
Kickxellomycetes
subphylum
Pezizo-mycotina
Saccharo-mycotina
Taphrino-mycotina
Mucoro-mycotina
Entomophthoro-mycotina
Kickxello-mycotina
phylum
Ascomycota
Mucoromycota
Zoopagomycota
Fungi
B
8 4 10 47 54 10 10 102 186 68 198
% of organisms
paralogs per organism
7 6 5 4 3 2 1
Po Pyronema omphalodes
Sc Saccharomyces cerevisiae
Cg Candida glabrata
Sc Smittium culicis
Sp Schizosaccharomyces pombe
Ca Candida albicans
Kp Komagataella pastoris
Kl Kluyveromyces lactis
Sa Smittium angustum
Yl Yarrowia lipolytica
Sp Spathaspora passalidarum
Sk Saccharomyces kudriavzevii
Mc Morchella conica
Cl Coniochaeta ligniaria
Af Aspergillus fumigatus
Nf Neosartorya fischeri
Ts Talaromyces stipitatus
Bg Blumeria graminis
Bc Botrytis cinerea
Fo Fusarium oxysporum
Mo Magnaporthe oryzae
Oo Orbilia oligospora
Tm Tuber melanosporum
Bo Bipolaris oryzae
Bd Blastomyces dermatitidis
Harpellomycetes
Schizosaccharomycetes
Dipodascomycetes
Pichiomycetes
Saccharomycetes
Orbiliomycetes
Pezizomycetes
Dothideomycetes
Eurotiomycetes
Leotiomycetes
Sordariomycetes
C
paralogs per organism
group I
group II
Harpellomycetes
Schizosaccharomycetes
Dipodascomycetes
Pichiomycetes
Saccharomycetes
Orbiliomycetes
Pezizomycetes
Dothideomycetes
Eurotiomycetes
Leotiomycetes
Sordariomycetes
2.5 2.0 1.5 1.0 0.5 0
D
# of paralogs
group I 1 1 1 1 2 2 2 2 3 4 4 4 - - -
group II - 1 2 6 - 1 2 3 1 - 1 2 1 2 3
Harpellomycetes
Schizosaccharomycetes
Dipodascomycetes
Pichiomycetes
Saccharomycetes
Orbiliomycetes
Pezizomycetes
Dothideomycetes
Eurotiomycetes
Leotiomycetes
Sordariomycetes
100 80 60 40 20 0

Figure S2. Presence and distribution of SUN domain protein in different fungi. A) Distribution of 1408 paralogous SUN domain proteins covering group I and II in a total of 710 fungal organisms corresponding to 607 fungal species (Table S1). Distribution patterns are shown in different colors according to their presence in eight taxonomic classes from the subphylum of Pezizomycotina (red), the class of Saccharomycetes from Saccharomycotina (orange), four classes of Taphrinomycotina (yellow), the class of Endogonomycetes from Mucoromycotina (purple), one class from Entomophthoromycotina (moss green), and two classes of the subphylum of Kickxellomycotina (light green), respectively. B) Percentage of fungal organisms carrying a given number of paralogous SUN domain proteins within nine ascomycetal classes. The number of paralogous SUN domain proteins per organism was determined for a total of 698 different ascomycetes (Table S1) covering nine different classes, followed by calculation of the percentage of organisms carrying a given number of paralogs (up to seven) for each class. Distribution bars show the percentage of organisms in each class in different colors according to the number of paralogs per organism. The absolute number of species analyzed in each class is indicated at the top of the distribution bars. 21 selected species are indicated in the respective bar segments in white letters using the abbreviations specified in the list on the right. Phylogeny of the nine ascomycetal classes is indicated by the tree shown at the bottom. C) Heatmap showing the average number of group I and group II SUN domain paralogs per organism in nine different ascomycetal classes shown in *B*. Complete absence of group I or group II paralogs is indicated by a grey slash. D) Heatmap showing the distribution patterns for group I and group II paralogs present in the nine different ascomycetal classes shown in *B* and *C*. The numbers of group I and group II paralogs present in the fifteen different distribution patterns uncovered is indicated on top. Complete absence of a given distribution pattern is indicated by a grey slash.

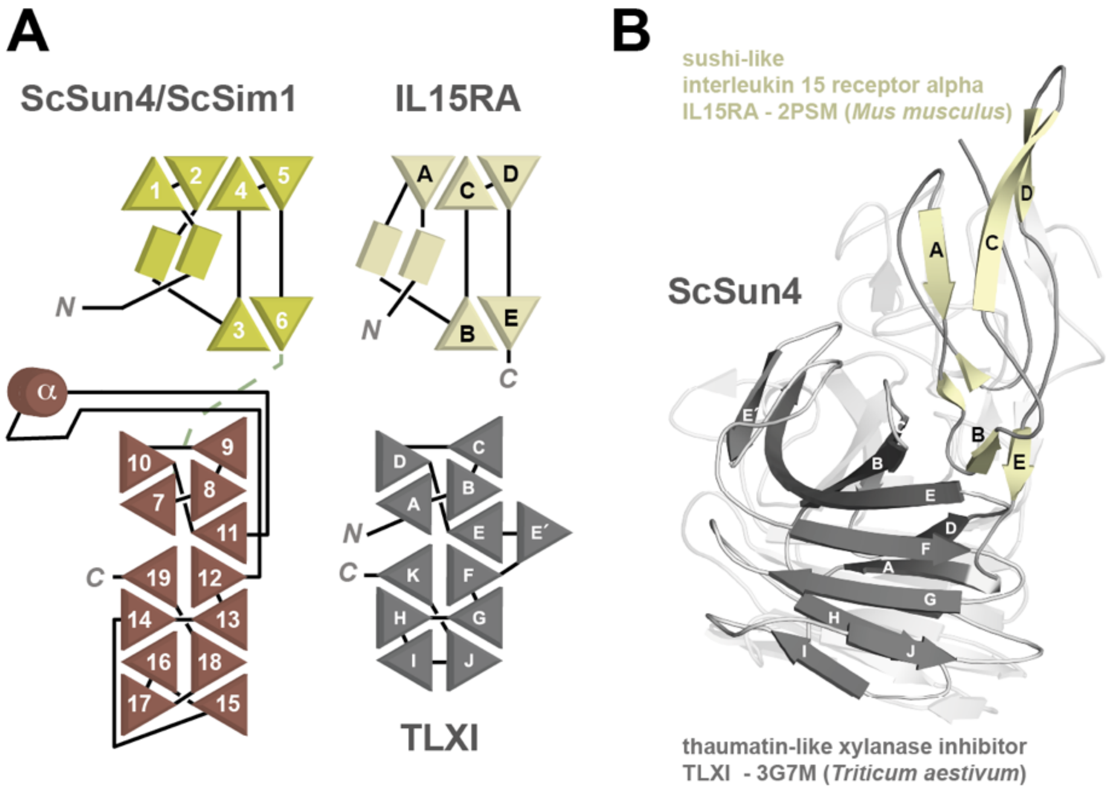

Figure S3. Structural topology of SUN domains from ScSun4 and ScSim1. A) Structural topology of the sushi-like (yellow) and thaumatin-like (brown) folds of ScSun4 and ScSim1 compared to the topologically related sushi-like fold of the interleukin 15 receptor α-subunit IL-15RA from *Mus musculus* (light yellow; PDB entry 2PSM) and the thaumatin-like xylanase inhibitor TLXI from *Triticum aestivum* (grey; PDB entry 3G7M), respectively. B) Structural alignment of the ScSun4 SUN domain (light grey) with IL-15RA (light yellow) and TLXI (grey) superimposed and calculated with PDBeFold. The sushi-like fold of IL-15RA aligns with a root mean square deviation of 2.57 Å for 54 C_α-atoms with the sushi domain of ScSun4, while TLXI matches for 120 C_α-atoms with a root mean square deviation of 2.64 Å.

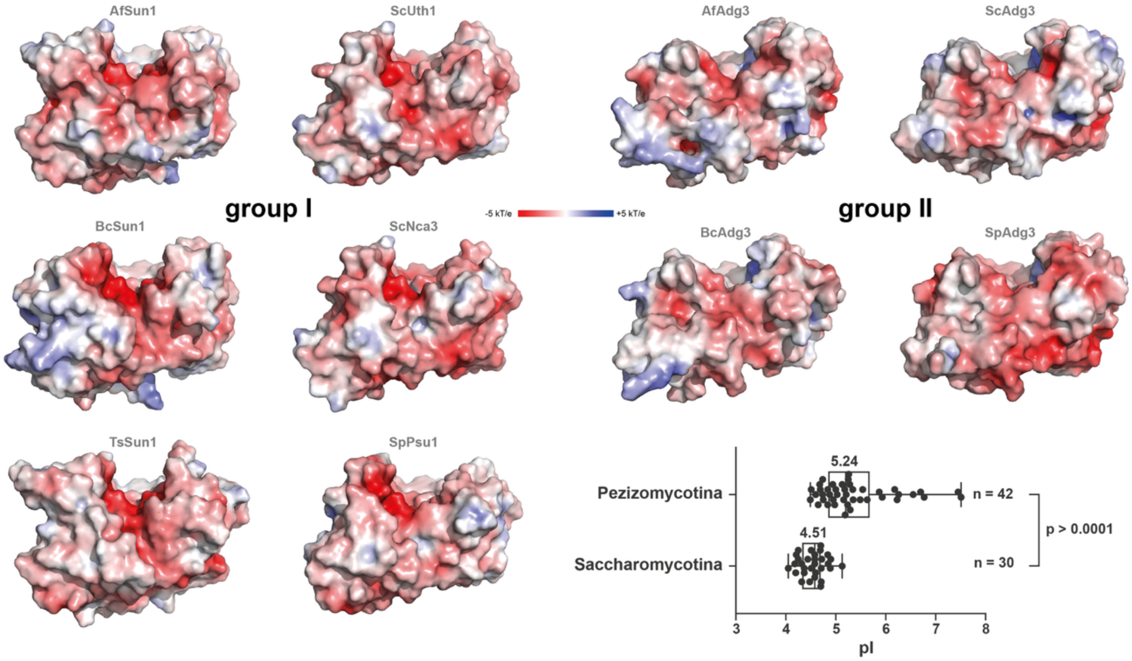

Figure S4. Surface charge and isoelectric points of fungal SUN domains. The surface charges of selected group I and group II SUN domains from the subphyla of Pezizomycotina (AfSun1, AfAdg3, BcSun1, BcAdg3, TsSun1), Saccharomycotina (ScUth1, ScNca3, ScAdg3) and Taphrinomycotina (SpPsu1, SpAdg3), which were functionally investigated this study, are color-coded as indicated and are based on structural models generated by AlphaFold (2). The diagram shows the comparison of the pI values calculated for 72 selected SUN domains from Pezizomycotina (n = 42) and Saccharomycotina (n = 30) that were used for the phylogenetic ML-tree analysis presented in Figure 1B. The individual pI values were calculated by ProtParam (3) and analyzed by a one sample t and Wilcoxon test. The median pI values calculated for Pezizomycotina (5.24) and Saccharomycotina (4.51) are indicated and significantly differ from each other ($p < 0.0001$).

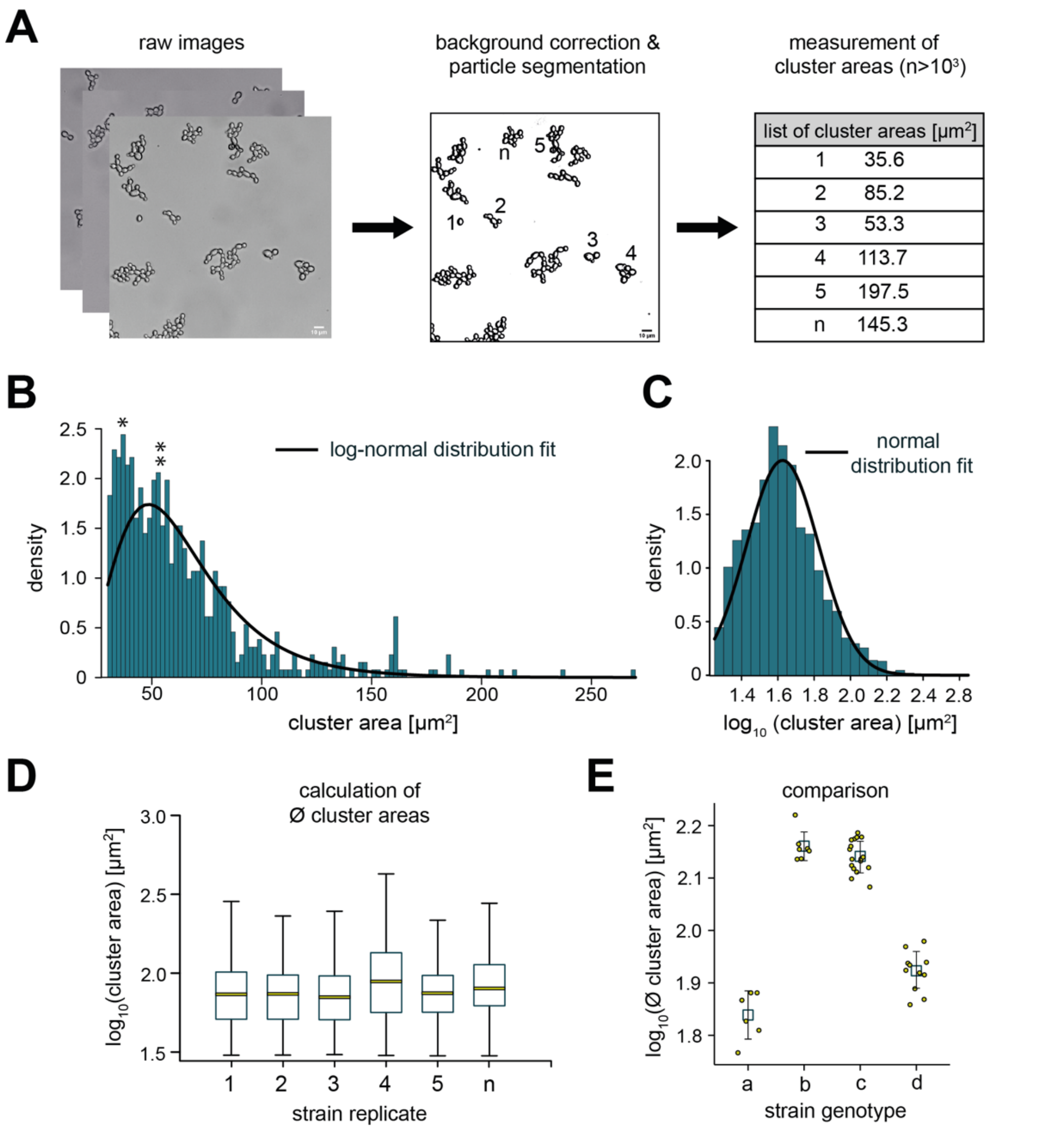

Figure S5. Quantitative cell cluster analysis (QCA) in *S. cerevisiae*. A) Image acquisition and processing. Images of yeast strains were obtained by low magnification (200x) brightfield microscopy and digital photography after growth of strains in liquid YNB cultures into early stationary phase and vigorous stirring by a vortex shaker. Digital raw images were further processed by the *ImageJ* software program (4) for background correction and particle segmentation prior to determination of individual cell cluster areas in μm^2. Routinely, the sizes of more than 1'000 random particles ($n>10^3$) were determined for each strain. Numbers indicate examples for a single cell (1) or for cell clusters with different sizes (2 - n). B) Histogram of cell cluster areas obtained for a wild type yeast strain (YHUM3154) and corresponding curve (black line) of a log-normal distribution fit. Subpopulations corresponding to single non-dividing or dividing cells are marked by single (*) or double (⁑) asterisks, respectively. C) Histogram of log_{10}-transformed data from *B)* and corresponding curve of a normal distribution fit. D) Distribution of cell cluster sizes (black bars) and calculated mean cell cluster areas (yellow lines in boxes) for independent measurements (1 - n) of a given yeast strain. E) Comparative presentation of independently measured mean cluster area values (yellow dots) and corresponding average values (small squares) together with standard deviations (black bars) for four yeast strains with different genotypes (a-d). B-E) Statistical analysis and visualization of data were performed by the *Microsoft Excel* and *R* software programs.

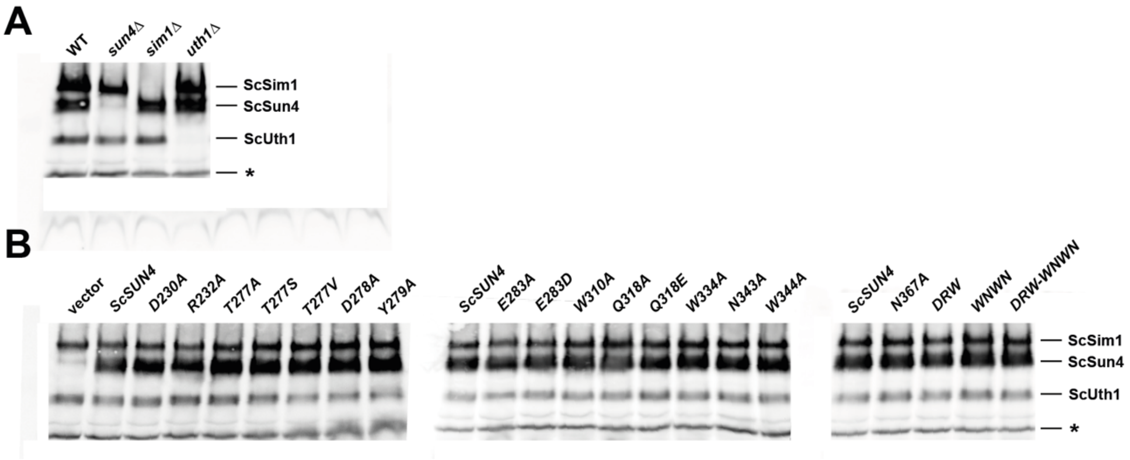

Figure S6. Expression of SUN domain proteins in *S. cerevisiae*. A) Expression of ScSun4, ScSim1 and ScUth1 in a control yeast strain (WT) and isogenic strains carrying the indicated chromosomal deletions of *ScSUN4* (*sun4*Δ), *ScSIM1* (*sim1*Δ) or *ScUTH1* (*uth1*Δ). Total protein extracts from yeast strains grown to exponential growth phase were analyzed by Western-blot using polyclonal antibodies raised against the recombinant SUN domain of ScSun4. The bands corresponding to ScSun4, ScSim1 and ScUth1, respectively, are indicated. An unknown protein is indicated by an asterisk. B) Expression of ScSun4 mutational variants. Yeast strains with a chromosomal *sun4*Δ mutation and carrying either a control plasmid (vector) or a plasmid with *ScSUN4* or a plasmid with the indicated *ScSUN4* mutational variant, respectively, were analyzed by Western blot as described in A). For comparison with the functional analysis of *ScSUN4* mutants see Figure 3D.

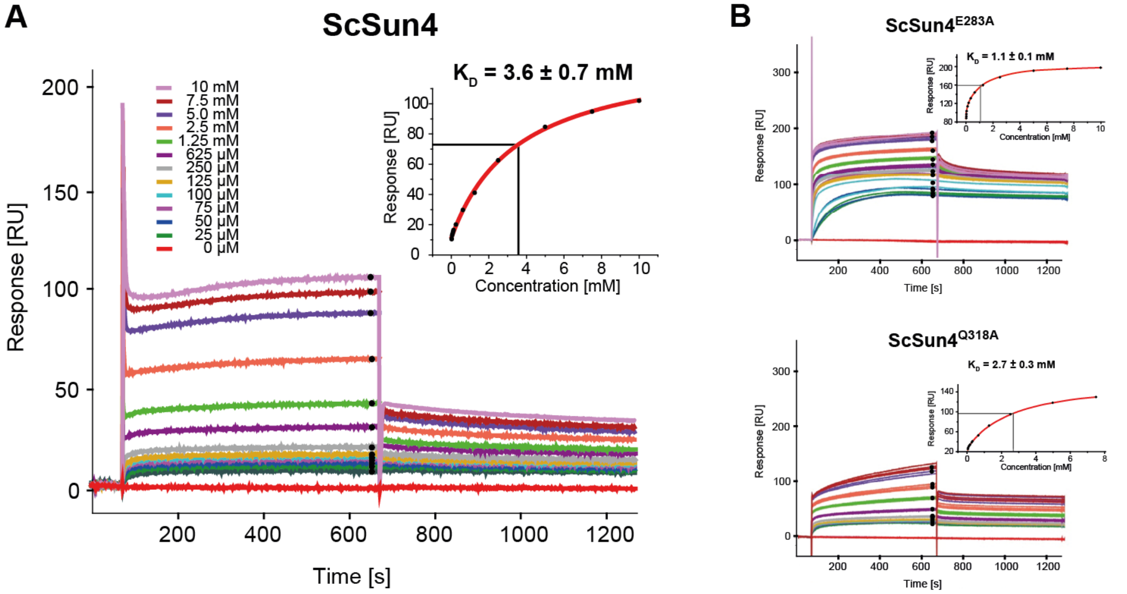

Figure S7. *In vitro* binding of ScSun4 and Laminarin. A) The biomolecular interaction between ScSun4 and laminarin was measured by surface plasmon resonance (SPR) spectroscopy at 20°C for approx. 600 s using immobilized ScSun4 SUN domain and increasing concentrations of laminarin varying in dense polymerisation (DP) ranging from 25 µM up to 10 mM. The binding constant shown (K_D = 3.6 mM) was then calculated under the assumption that the average molecular weight of the Laminarin used corresponds to 4066 Da. B) Constants for laminarin-binding of ScSun4^{E283A} (K_D = 1.1 mM) and ScSun4^{Q318A} (K_D = 2.7 mM), respectively, were determined as described in A).

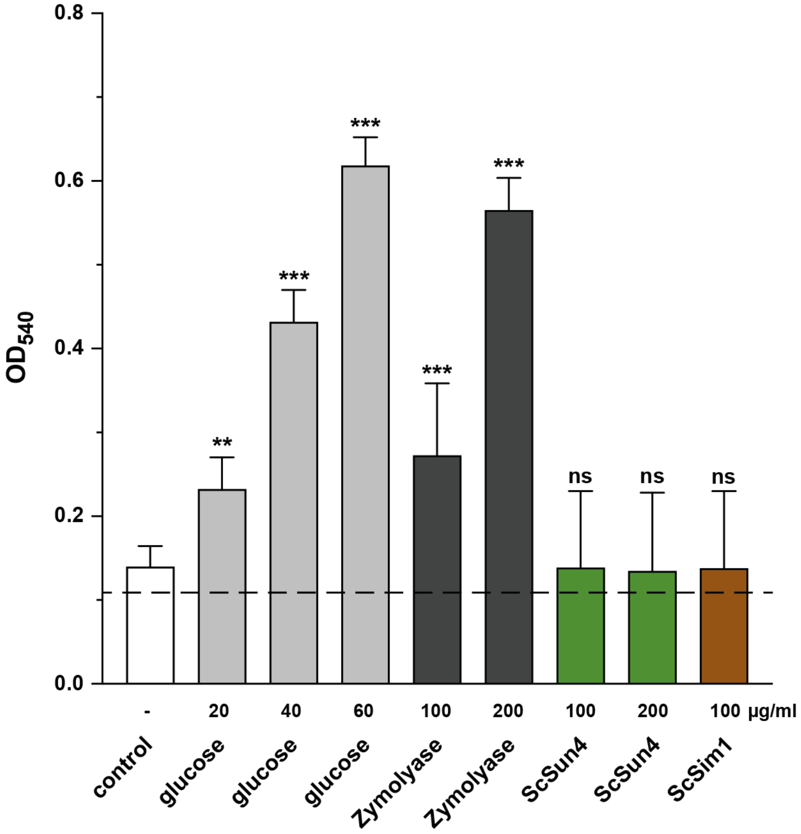

Figure S8. *In vitro* glucanase activity of ScSun4 and ScSim1. Glucanase activity towards laminarin of ScSun4 and ScSim1 was measured using the respective recombinant SUN domains at the indicated concentrations. As a known glucanase, Zymolyase 100T (Zymolyase) was used. Glucose was used as a control for the quantification of reducing sugars, that result from degradation of laminarin and react with 3,5-dinitrosalicylic acid resulting in 3-amino-5-nitrosalicylic acid, which can be detected at OD_{540}. Statistical significance of the differences measured in comparison to a control using only water (first bar) was calculated by an unpaired t test and is indicated according to the P values obtained: ns for P values > 0.05; ** for P values between 0.005 and 0.0005; *** for P values < 0.0005. The dotted line indicates the OD_{540} obtained in the absence of laminarin.

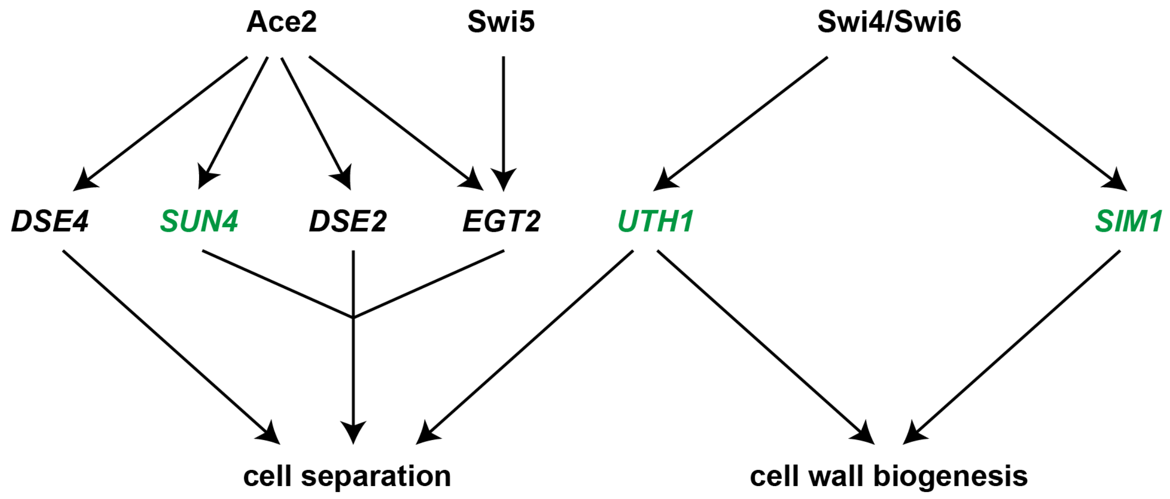

Figure S9. Model for control of *S. cerevisiae* cell separation and cell wall biogenesis by SUN domain family members *SUN4*, *UTH1*, *SIM1*, and by *DSE2*, *DSE4* and *EGT2*.
Regulation of *DSE4*, *SUN4*, *DSE2* and *EGT2* by the transcription factors Ace2 and Swi5 has been reported (5-7). Control of cell separation by *DSE4*, *SUN4*, *DSE2* and *EGT2* has been reviewed (8) and is supported by the data presented in Figure 4. Existence of a Sun4-Dse2-Egt2 protein complex is supported by a previous co-localization study (9) and by genetic interaction analysis shown in Figure 4. Regulation of *UTH1* and *SIM1* by the transcription factors Swi4/Swi6 is supported by previous studies (6, 10, 11). Involvement of *UTH1* and *SIM1* in cell wall biogenesis is supported by previous genetic analysis (12). SUN domain family members are shown in green.

Supplemental Tables S2 to S8

Table S2. Data collection and refinement statistics.

	**Sun4 (9T47)**	**Sun4^{E283A} (9T4O)**	**Sun4^{Q318A} (9T4N)**	**Sim1 (9T4Q)**
Wavelength (Å), beamline	1.0332 (ID14-4)	0.9184 (MX14-1)	0.9184 (MX14-1)	1.0000 (X06SA)
Resolution range (Å)	36.72-1.05 (1.11-1.05)	36.37-1.24 (1.30-1.24)	36.37-1.28 (1.35-1.28)	24.9-1.23 (1.27-1.23)
Space group	*I*222	*I*222	*I*222	$P2_12_12_1$
Unit cell (Å)	62.4 99.2 102.2	62.33 99.09 101.53	62.4 99.2 102.2	41.04 78.64 80.20
Total reflections	1081470	550640	542861	496336 (49260)
Unique reflections	143630	88813	81024	74828 (7201)
Multiplicity	7.5 (3.4)	6.2 (6.0)	6.7 (6.5)	6.6 (6.8)
Completeness (%)	97.7 (85.8)	99.8 (97.9)	100 (100)	98.3 (95.9)
Mean I/sigma(I)	19.9 (2.5)	13.8 (2.7)	11.8 (3.1)	14.52 (1.5)
Wilson *B*-factor (Å^2)	12.1	21.4	19.5	17.8
R_{merge} (%)	4.9 (53.5)	4.9 (64.0)	4.9 (55.5)	6.2 (155)
Reflections used in refinement	119598 (11649)	87901 (8619)	80942 (8041)	74796 (7197)
Reflections used for R_{free}	7975 (797)	5510 (571)	5055 (482)	3583 (376)
R_{work}	0.1232 (0.2019)	0.1302 (0.2322)	0.1411 (0.2837)	0.1452 (0.3822)
R_{free}	0.1416 (0.2204)	0.1554 (0.2553)	0.1684 (0.2979)	0.1664 (0.4461)
CC_{work}	0.959 (0.877)	0.976 (0.877)	0.973 (0.747)	0.973 (0.849)
CC_{free}	0.944 (0.843)	0.974 (0.866)	0.972 (0.724)	0.963 (0.809)
Number of non-H atoms	2627	2647	2601	2644
macromolecules	2172	2235	2214	2233
ligands	45	55	46	1
solvent	436	386	367	410

Protein residues	282	282	282	287
RMS – bonds (Å)	0.008	0.007	0.005	0.007
RMS – angles (°)	1.04	0.97	0.86	1.01
Ramachandran favoured (%)	97.86	97.86	98.21	98.23
Ramachandran allowed (%)	2.14	2.14	1.79	1.77
Ramachandran outliers (%)	0.00	0.00	0.00	0.00
Rotamer outliers (%)	0.41	0.39	1.57	1.19
Clashscore	3.07	2.48	3.44	5.08
Average *B*-factor (Å^2)	18.5	21.1	20.6	23.5
macromolecules (Å^2)	15.7	18.8	18.4	21.4
ligands (Å^2)	39.0	42.2	40.5	63.3
solvent (Å^2)	31.3	33.1	32.5	35.1

Statistics for the highest-resolution shell are shown in parentheses

Table S3. Statistical significance for comparative analysis of effects of mutations in *S. cerevisiae SUN4* on *S. cerevisiae* cell clustering (data shown in Figure 3B).

Strain	Genotype[1]	Plasmid[2]	Av $\log_{10}$ (Ø cluster area)[3]	P value[4] for comparison to strain 1	P value[4] for comparison to strain 2
1	*SUN4*	-	1.84	-	<0.001
2	*sun4*Δ	-	2.16	<0.001	-
3	*sun4*Δ	control	2.14	<0.001	0.18
4	*sun4*Δ	*ScSUN4*	1.92	0.004	<0.001

[1]*S. cerevisiae* chromosomal genotype (homozygous diploid; Table S7)
[2]*ScSUN4* variant expressed from plasmid (Table S8)
[3]Values correspond to the average values of the $\log_{10}$ (mean cluster area) data points presented in Figure 3B.
[4]P values were calculated applying an unpaired t-test using the data presented in Figure 3B.

Table S4. Statistical significance for comparative analysis of effects of mutations in *ScSUN4* on *S. cerevisiae* cell clustering (data shown in Figure 3D).

Strain	Genotype[1]	Plasmid[2]	Av $\log_{10}$ (Ø cluster area)[3]	P value[4] for comparison to strain 1	P value[4] for comparison to strain 2
1	*sun4*Δ	control	2.14	-	<0.001
2	*sun4*Δ	*ScSUN4*	1.92	<0.001	-
3	*sun4*Δ	*ScSUN4*^{D230A}	1.87	<0.001	0.02
4	*sun4*Δ	*ScSUN4*^{R232A}	1.88	<0.001	0.14
5	*sun4*Δ	*ScSUN4*^{T277A}	2.06	0.007	<0.001
6	*sun4*Δ	*ScSUN4*^{T277S}	2.05	<0.001	<0.001
7	*sun4*Δ	*ScSUN4*^{T277V}	1.92	<0.001	0.95
8	*sun4*Δ	*ScSUN4*^{D278A}	1.99	<0.001	0.002
9	*sun4*Δ	*ScSUN4*^{Y279A}	1.98	<0.001	0.007
10	*sun4*Δ	*ScSUN4*^{E283A}	2.12	0.22	<0.001
11	*sun4*Δ	*ScSUN4*^{E283D}	1.94	<0.001	0.24
12	*sun4*Δ	*ScSUN4*^{W310A}	1.88	<0.001	0.38
13	*sun4*Δ	*ScSUN4*^{Q318A}	2.04	0.008	0.004
14	*sun4*Δ	*ScSUN4*^{Q318E}	2.06	0.009	<0.001
15	*sun4*Δ	*ScSUN4*^{W334A}	1.91	<0.001	0.75
16	*sun4*Δ	*ScSUN4*^{N343A}	1.87	<0.001	0.02
17	*sun4*Δ	*ScSUN4*^{W344A}	1.88	<0.001	0.12
18	*sun4*Δ	*ScSUN4*^{N367A}	1.87	<0.001	0.03
19[5]	*sun4*Δ	*ScSUN4*^{DRW}	2.05	<0.001	<0.001
20[6]	*sun4*Δ	*ScSUN4*^{WNWN}	2.13	0.47	<0.001
21[7]	*sun4*Δ	*ScSUN4*$^{DRW-WNWN}$	2.14	0.53	<0.001

[1]*S. cerevisiae* chromosomal genotype (homozygous diploid; Table S7)
[2]*ScSUN4* variant expressed from plasmid (Table S8)
[3]Values correspond to the average values of the $\log_{10}$ (mean cluster area) data points presented in Figure 3D.
[4]P values were calculated applying an unpaired t-test using the data presented in Figure 3D.
[5]*ScSUN4*^{DRW} corresponds to *ScSUN4*$^{D230A\ R232A\ W310A}$
[6]*ScSUN4*^{WNWN} corresponds to *ScSUN4*$^{W334A\ N343A\ W344A\ N367A}$
[7]*ScSUN4*$^{DRW-WNWN}$ corresponds to *ScSUN4*$^{D230A\ R232A\ W310A\ W334A\ N343A\ W344A\ N367A}$

Table S5. Statistical significance for comparative analysis of effects of mutations in *S. cerevisiae SUN4*, *SIM1, UTH1, NCA3*, *ADG3, DSE2, EGT2, ACE2 and SWI5* on *S. cerevisiae* cell clustering (data shown in Figure 4).

Strain	Genotype[1]	Av $\log_{10}$ (Ø cluster area)[2]	P value[3] for comparison to strain 1	P value[3] for comparison to strain 2
1	*wt*	1.84	-	<0.001
2	*sun4Δ*	2.16	<0.001	-
3	*sim1Δ*	1.85	0.73	<0.001
4	*uth1Δ*	1.88	0.02	<0.001
5	*nca3Δ*	1.82	0.06	<0.001
6	*adg3Δ*	1.87	0.11	<0.001
7	*sun4Δ sim1Δ*	2.14	<0.001	0.23
8	*sun4Δ uth1Δ*	2.58	<0.001	<0.001
9	*sun4Δ nca3Δ*	2.18	<0.001	0.11
10	*sun4Δ adg3Δ*	2.17	<0.001	0.15
11	*dse2Δ*	2.20	<0.01	0.67
12	*egt2Δ*	2.14	<0.01	0.65
13	*sun4Δ dse2Δ*	2.19	<0.01	0.10
14	*sun4Δ egt2Δ*	2.22	<0.001	0.18
15	*dse2Δ egt2Δ*	2.16	<0.01	0.96
16	*sun4Δ dse2Δ egt2Δ*	2.20	<0.001	0.33
17	*ace2Δ*	2.51	<0.001	<0.001
18	*swi5Δ*	2.15	<0.001	0.78
19	*ace2Δ swi5Δ*	2.66	<0.001	<0.001

[1]*S. cerevisiae* chromosomal genotype (homozygous diploid; Table S7)
[2]Values correspond to the average values of the $\log_{10}$ (mean cluster area) data points presented in Figure 4.
[3]P values were calculated applying an unpaired t-test using the data presented in Figure 4.

Table S6. Statistical significance for comparative analysis of effects of *ScSUN4*-chimeras on *S. cerevisiae* cell clustering (data shown in Figure 5B).

Strain	Genotype[1]	Plasmid[2]	Av $\log_{10}$ (Ø cluster area)[3]	P value[4] for comparison to strain 1	P value[4] for comparison to strain 2
1	*sun4Δ*	control	2.14	-	<0.001
2	*sun4Δ*	*ScSUN4*	1.92	<0.001	-
3	*sun4Δ*	*ScSUN4*^{T277A}	2.06	0.007	<0.001
4	*sun4Δ*	*ScSUN4*^{E283A}	2.12	0.22	<0.001
5	*sun4Δ*	*ScSUN4-ScSIM1*	1.94	<0.001	0.34
6	*sun4Δ*	*ScSUN4-ScSIM1*^{T333A}	2.11	0.03	<0.001
7	*sun4Δ*	*ScSUN4-ScSIM1*^{E339A}	2.15	0.06	<0.001
8	*sun4Δ*	*ScSUN4-ScUTH1*	1.95	<0.001	0.15
9	*sun4Δ*	*ScSUN4-ScNCA3*	2.06	<0.001	<0.001
10	*sun4Δ*	*ScSUN4-ScADG3*	2.09	0.008	<0.001
11	*sun4Δ*	*ScSUN4-SpPSU1*	1.94	<0.001	0.23
12	*sun4Δ*	*ScSUN4-SpPSU1*^{T276A}	2.03	<0.001	<0.001
13	*sun4Δ*	*ScSUN4-SpPSU1*^{E282A}	2.12	0.37	<0.001
14	*sun4Δ*	*ScSUN4-SpADG3*	2.14	0.59	<0.001
15	*sun4Δ*	*ScSUN4-AfSUN1*	1.96	<0.001	0.009
16	*sun4Δ*	*ScSUN4-AfADG3*	2.12	0.09	<0.001
17	*sun4Δ*	*ScSUN4-TsSUN1*	2.11	0.03	<0.001
18	*sun4Δ*	*ScSUN4-BcSUN1*	2.13	0.44	<0.001
19	*sun4Δ*	*ScSUN4-BcADG3*	2.15	0.08	<0.001

[1] *S. cerevisiae* chromosomal genotype (homozygous diploid; Table S7)
[2] *ScSUN4* chimeric variant expressed from plasmid (Table S8)
[3] Values correspond to the average values of the $\log_{10}$ (mean cluster area) data points presented in Figure 5B.
[4] P values were calculated applying an unpaired t-test using the data presented in Figure 5B.

Table S7. Yeast strains used in this study

Strain	Relevant genotype	Source
YHUM470	*MAT***a** *ura3Δ leu2Δ his3Δ*	Microbia Inc.
YHUM471	*MATα ura3Δ leu2Δ his3Δ*	Microbia Inc.
YHUM3107	*MAT***a** *sun4Δ::hphNT1 ura3Δ leu2Δ his3Δ*	This work
YHUM3108	*MATα sun4Δ::hphNT1 ura3Δ leu2Δ his3Δ*	This work
YHUM3101	*MAT***a** *sim1Δ::kanMX6 ura3Δ leu2Δ his3Δ*	This work
YHUM3103	*MATα sim1Δ::kanMX6 ura3Δ leu2Δ his3Δ*	This work
YHUM3104	*MAT***a** *uth1Δ::natNT2 ura3Δ leu2Δ his3Δ*	This work
YHUM3106	*MATα uth1Δ::natNT2 ura3Δ leu2Δ his3Δ*	This work
YHUM2881	*MAT***a** *nca3Δ::kanMX6 ura3Δ leu2Δ his3Δ*	This work
YHUM2883	*MATα nca3Δ::kanMX6 ura3Δ leu2Δ his3Δ*	This work
YHUM3605	*MAT***a** *adg3Δ::kanMX6 ura3Δ leu2Δ his3Δ*	This work
YHUM3607	*MATα adg3Δ::kanMX6 ura3Δ leu2Δ his3Δ*	This work
YHUM3166	*MAT***a** *dse2Δ::kanMX4 ura3Δ leu2Δ his3Δ*	This work
YHUM3167	*MATα dse2Δ::kanMX4 ura3Δ leu2Δ his3Δ*	This work
YHUM3220	*MAT***a** *egt2Δ::natNT2 ura3Δ leu2Δ his3Δ*	This work
YHUM3221	*MATα egt2Δ::natNT2 ura3Δ leu2Δ his3Δ*	This work
YHUM3208	*MAT***a** *ace2Δ::kanMX6 ura3Δ leu2Δ his3Δ*	This work
YHUM3209	*MATα ace2Δ::kanMX6 ura3Δ leu2Δ his3Δ*	This work
YHUM3212	*MAT***a** *swi5Δ::kanMX6 ura3Δ leu2Δ his3Δ*	This work
YHUM3213	*MATα swi5Δ::kanMX6 ura3Δ leu2Δ his3Δ*	This work
YHUM3154	*MAT***a**/*MATα ura3Δ/ura3Δ leu2Δ/leu2Δ his3Δ/ his3Δ*	This work
YHUM3160	*MAT***a**/*MATα sun4Δ::hphNT1/sun4Δ::hphNT1 ura3Δ/ura3Δ leu2Δ/leu2Δ his3Δ/ his3Δ*	This work
YHUM3156	*MAT***a**/*MATα sim1Δ::kanMX6/sim1Δ::kanMX6 ura3Δ/ura3Δ leu2Δ/leu2Δ his3Δ/ his3Δ*	This work
YHUM3115	*MAT***a**/*MATα uth1Δ::natNT2/uth1Δ::natNT2 ura3Δ/ura3Δ leu2Δ/leu2Δ his3Δ/ his3Δ*	This work
YHUM3602	*MAT***a**/*MATα nca3Δ::kanMX6/nca3Δ::kanMX6 ura3Δ/ura3Δ leu2Δ/leu2Δ his3Δ/ his3Δ*	This work
YHUM3630	*MAT***a**/*MATα adg3Δ::kanMX6/adg3Δ::kanMX6 ura3Δ/ura3Δ leu2Δ/leu2Δ his3Δ/ his3Δ*	This work
YHUM3162	*MAT***a**/*MATα sun4Δ::hphNT1/sun4Δ::hphNT1 sim1Δ::kanMX6/sim1Δ::kanMX6 ura3Δ/ura3Δ leu2Δ/leu2Δ his3Δ/ his3Δ*	This work
YHUM3164	*MAT***a**/*MATα sun4Δ::hphNT1/sun4Δ::hphNT1 uth1Δ::natNT2/uth1Δ::natNT2 ura3Δ/ura3Δ leu2Δ/leu2Δ his3Δ/ his3Δ*	This work
YHUM3642	*MAT***a**/*MATα sun4Δ::hphNT1/sun4Δ::hphNT1 nca3Δ::kanMX6/nca3Δ::kanMX6*	This work

	ura3Δ/ura3Δ leu2Δ/leu2Δ his3Δ/ his3Δ	
YHUM3637	*MAT***a***/MATα sun4Δ::hphNT1/sun4Δ::hphNT1 adg3Δ::kanMX6/adg3Δ::kanMX6 ura3Δ/ura3Δ leu2Δ/leu2Δ his3Δ/ his3Δ*	This work
YHUM3168	*MAT***a***/MATα dse2Δ::kanMX4/dse2Δ::kanMX4 ura3Δ/ura3Δ leu2Δ/leu2Δ his3Δ/ his3Δ*	This work
YHUM3170	*MAT***a***/MATα egt2Δ::natNT2/egt2Δ::natNT2 ura3Δ/ura3Δ leu2Δ/leu2Δ his3Δ/ his3Δ*	This work
YHUM3224	*MAT***a***/MATα sun4Δ::hphNT1/sun4Δ::hphNT1 dse2Δ::kanMX4/dse2Δ::kanMX4 ura3Δ/ura3Δ leu2Δ/leu2Δ his3Δ/ his3Δ*	This work
YHUM3232	*MAT***a***/MATα sun4Δ::hphNT1/sun4Δ::hphNT1 egt2Δ::natNT2/egt2Δ::natNT2 ura3Δ/ura3Δ leu2Δ/leu2Δ his3Δ/ his3Δ*	This work
YHUM3228	*MAT***a***/MATα dse2Δ::kanMX4/dse2Δ::kanMX4 egt2Δ::natNT2/egt2Δ::natNT2 ura3Δ/ura3Δ leu2Δ/leu2Δ his3Δ/ his3Δ*	This work
YHUM3236	*MAT***a***/MATα sun4Δ::hphNT1/sun4Δ::hphNT1 dse2Δ::kanMX4/dse2Δ::kanMX4 egt2Δ::natNT2/egt2Δ::natNT2 ura3Δ/ura3Δ leu2Δ/leu2Δ his3Δ/ his3Δ*	This work
YHUM3210	*MAT***a***/MATα ace2Δ::kanMX6/ace2Δ::kanMX6 ura3Δ/ura3Δ leu2Δ/leu2Δ his3Δ/ his3Δ*	This work
YHUM3214	*MAT***a***/MATα swi5Δ::kanMX6/swi5Δ::kanMX6 ura3Δ/ura3Δ leu2Δ/leu2Δ his3Δ/ his3Δ*	This work
YHUM3218	*MAT***a***/MATα ace2Δ::kanMX6/ace2Δ::kanMX6 swi5Δ::kanMX6/swi5Δ::kanMX6 ura3Δ/ura3Δ leu2Δ/leu2Δ his3Δ/ his3Δ*	This work

Table S8. Plasmids used in this study

Plasmid	Relevant genotype	Source
pFA6a-kanMX6	*kanMX6*	(13)
pFA6a-hphNT1	*hphNT1*	(14)
pFA6a-natNT2	*natNT2*	(14)
pET-28a(+)	*kanR* P_{T7} *6xHis lacI*	Merck, Germany
pET-28a-*ScSUN4*	*ScSUN4*$^{G147-N420}$ in pET-28a(+)	This work
pET-28a-*ScSUN4*^{E283A}	*ScSUN4*$^{G147-N420,E283A}$ in pET-28a(+)	This work
pET-28a-*ScSUN4*^{Q318A}	*ScSUN4*$^{G147-N420,Q318A}$ in pET-28a(+)	This work
BHUM3442	*ScSIM1*$^{G202-N476}$ in pET-28a(+)	This work
BHUM3454	*SpPSU1*$^{G165-Y417}$ in pET-28a(+)	This work
pRS316	*CEN URA3*	(15)
BHUM3438	*ScSUN4* in pRS316	This work
BHUM3657	*ScSUN4*^{D230A} in pRS316	This work
BHUM3658	*ScSUN4*^{R232A} in pRS316	This work
BHUM3659	*ScSUN4*^{T277A} in pRS316	This work
BHUM3661	*ScSUN4*^{T277S} in pRS316	This work
BHUM3662	*ScSUN4*^{T277V} in pRS316	This work
BHUM3663	*ScSUN4*^{D278A} in pRS316	This work
BHUM3664	*ScSUN4*^{Y279A} in pRS316	This work
BHUM3665	*ScSUN4*^{E283A} in pRS316	This work
BHUM3666	*ScSUN4*^{E283D} in pRS316	This work
BHUM3667	*ScSUN4*^{W310A} in pRS316	This work
BHUM3668	*ScSUN4*^{Q318A} in pRS316	This work
BHUM3669	*ScSUN4*^{Q318E} in pRS316	This work
BHUM3670	*ScSUN4*^{W334A} in pRS316	This work
BHUM3671	*ScSUN4*^{N343A} in pRS316	This work
BHUM3672	*ScSUN4*^{W344A} in pRS316	This work
BHUM3680	*ScSUN4*^{N367A} in pRS316	This work
BHUM4052[1]	*ScSUN4*^{DRW} in pRS316	This work
BHUM4054[2]	*ScSUN4*^{WNWN} in pRS316	This work
BHUM4056[3]	*ScSUN4*$^{DRW-WNWN}$ in pRS316	This work
BHUM3446	*ScSUN4*$^{M1-Y150}$-*ScSIM1*$^{G207-Y476}$ in pRS316	This work
BHUM3676	*ScSUN4*$^{M1-Y150}$-*ScSIM1*$^{G207-Y476,T333A}$ in pRS316	This work

BHUM3677	*ScSUN4*$^{M1\text{-}Y150}$-*ScSIM1*$^{G207\text{-}Y476,E339A}$ in pRS316	This work
BHUM4093	*ScSUN4*$^{M1\text{-}Y150}$-*ScUTH1*$^{S101\text{-}Y365}$ in pRS316	This work
BHUM4063	*ScSUN4*$^{M1\text{-}Y150}$-*ScNCA3*$^{G79\text{-}Y337}$ in pRS316	This work
BHUM4043	*ScSUN4*$^{M1\text{-}Y150}$-*ScADG3*$^{G48\text{-}D318}$ in pRS316	This work
BHUM3444	*ScSUN4*$^{M1\text{-}Y150}$-*SpPSU1*$^{G156\text{-}N417}$ in pRS316	This work
BHUM3678	*ScSUN4*$^{M1\text{-}Y150}$-*SpPSU1*$^{G156\text{-}N417,T276A}$ in pRS316	This work
BHUM3679	*ScSUN4*$^{M1\text{-}Y150}$-*SpPSU1*$^{G156\text{-}N417,E282A}$ in pRS316	This work
BHUM4044	*ScSUN4*$^{M1\text{-}Y150}$-*SpADG3*$^{T70\text{-}D335}$ in pRS316	This work
BHUM4042	*ScSUN4*$^{M1\text{-}Y150}$-*AfSUN1*$^{T138\text{-}Y414}$ in pRS316	This work
BHUM4069	*ScSUN4*$^{M1\text{-}Y150}$-*AfADG3*$^{G51\text{-}E315}$ in pRS316	This work
BHUM4046	*ScSUN4*$^{M1\text{-}Y150}$-*TsSUN1*$^{G154\text{-}Y433}$ in pRS316	This work
BHUM4065	*ScSUN4*$^{M1\text{-}Y150}$-*BcSUN1*$^{S189\text{-}S471}$ in pRS316	This work
BHUM4067	*ScSUN4*$^{M1\text{-}Y150}$-*BcADG3*$^{G60\text{-}A322}$ in pRS316	This work

[1]*ScSUN4*^{DRW} corresponds to *ScSUN4*$^{D230A\ R232A\ W310A}$

[2]*ScSUN4*^{WNWN} corresponds to *ScSUN4*$^{W334\ \ N343A\ W344A\ N367A}$

[3]*ScSUN4*$^{DRW\text{-}WNWN}$ corresponds to *ScSUN4*$^{D230A\ R232A\ W310A\ W334\ \ N343A\ W344A\ N367A}$

Supplemental References

1. Madeira F, Park YM, Lee J, Buso N, Gur T, Madhusoodanan N, Basutkar P, Tivey ARN, Potter SC, Finn RD, Lopez R. 2019. The EMBL-EBI search and sequence analysis tools APIs in 2019. Nucleic Acids Res 47:W636-W641.
2. Varadi M, Anyango S, Deshpande M, Nair S, Natassia C, Yordanova G, Yuan D, Stroe O, Wood G, Laydon A, Zidek A, Green T, Tunyasuvunakool K, Petersen S, Jumper J, Clancy E, Green R, Vora A, Lutfi M, Figurnov M, Cowie A, Hobbs N, Kohli P, Kleywegt G, Birney E, Hassabis D, Velankar S. 2022. AlphaFold Protein Structure Database: massively expanding the structural coverage of protein-sequence space with high-accuracy models. Nucleic Acids Res 50:D439-D444.
3. Wilkins MR, Gasteiger E, Bairoch A, Sanchez JC, Williams KL, Appel RD, Hochstrasser DF. 1999. Protein identification and analysis tools in the ExPASy server. Methods Mol Biol 112:531-52.
4. Schneider CA, Rasband WS, Eliceiri KW. 2012. NIH Image to ImageJ: 25 years of image analysis. Nat Methods 9:671-5.
5. Hu Z, Killion PJ, Iyer VR. 2007. Genetic reconstruction of a functional transcriptional regulatory network. Nat Genet 39:683-7.
6. MacIsaac KD, Wang T, Gordon DB, Gifford DK, Stormo GD, Fraenkel E. 2006. An improved map of conserved regulatory sites for Saccharomyces cerevisiae. BMC Bioinformatics 7:113.
7. Mostovoy Y, Thiemicke A, Hsu TY, Brem RB. 2016. The Role of Transcription Factors at Antisense-Expressing Gene Pairs in Yeast. Genome Biol Evol 8:1748-61.
8. Weiss EL. 2012. Mitotic exit and separation of mother and daughter cells. Genetics 192:1165-202.
9. Kuznetsov E, Vachova L, Palkova Z. 2016. Cellular localization of Sun4p and its interaction with proteins in the yeast birth scar. Cell Cycle 15:1898-907.
10. Hughes TR, Marton MJ, Jones AR, Roberts CJ, Stoughton R, Armour CD, Bennett HA, Coffey E, Dai H, He YD, Kidd MJ, King AM, Meyer MR, Slade D, Lum PY, Stepaniants SB, Shoemaker DD, Gachotte D, Chakraburtty K, Simon J, Bard M, Friend SH. 2000. Functional discovery via a compendium of expression profiles. Cell 102:109-26.
11. Venters BJ, Wachi S, Mavrich TN, Andersen BE, Jena P, Sinnamon AJ, Jain P, Rolleri NS, Jiang C, Hemeryck-Walsh C, Pugh BF. 2011. A comprehensive genomic binding map of gene and chromatin regulatory proteins in Saccharomyces. Mol Cell 41:480-92.
12. Mouassite M, Guerin MG, Camougrand NM. 2000. The SUN family of Saccharomyces cerevisiae: the double knock-out of UTH1 and SIM1 promotes defects in nucleus migration and increased drug sensitivity. FEMS Microbiol Lett 182:137-41.
13. Wach A. 1996. PCR-synthesis of marker cassettes with long flanking homology regions for gene disruptions in S. cerevisiae. Yeast 12:259-65.
14. Janke C, Magiera MM, Rathfelder N, Taxis C, Reber S, Maekawa H, Moreno-Borchart A, Doenges G, Schwob E, Schiebel E, Knop M. 2004. A versatile toolbox for PCR-based tagging of yeast genes: new fluorescent proteins, more markers and promoter substitution cassettes. Yeast 21:947-62.
15. Sikorski RS, Hieter P. 1989. A system of shuttle vectors and yeast host strains designed for efficient manipulation of DNA in *Saccharomyces cerevisiae*. Genetics 122:19-27.
